# Ventricular volume in relation to lumbar CSF levels of amyloid-β 1–42, tau and phosphorylated tau in iNPH, is there a dilution effect?

**DOI:** 10.1186/s12987-022-00353-9

**Published:** 2022-07-17

**Authors:** Simon Lidén, Dan Farahmand, Katarina Laurell

**Affiliations:** 1 Department of Medical Sciences, Neurology, Uppsala University, Uppsala, Sweden; 2 Department of Clinical Neuroscience, Institute of Neuroscience and Physiology, Sahlgrenska Academy, Gothenburg University, Gothenburg, Sweden

**Keywords:** Normal Pressure Hydrocephalus, Ventricular volume, Amyloid-β, Tau, Cerebrospinal fluid

## Abstract

**Background:**

Levels of the biomarkers amyloid-β 1–42 (Aβ42), tau and phosphorylated tau (p-tau) are decreased in the cerebrospinal fluid (CSF) of patients with idiopathic normal pressure hydrocephalus (iNPH). The mechanism behind this is unknown, but one potential explanation is dilution by excessive CSF volumes. The aim of this study was to investigate the presence of a dilution effect, by studying the relationship between ventricular volume (VV) and the levels of the CSF biomarkers.

**Methods:**

In this cross-sectional observational study, preoperative magnetic resonance imaging (MRI) and lumbar CSF was acquired from 136 patients with a median age of 76 years, 89 men and 47 females, selected for surgical treatment for iNPH. The CSF volume of the lateral and third ventricles was segmented on MRI and related to preoperative concentrations of Aβ42, tau and p-tau.

**Results:**

In the total sample VV (Median 140.7 mL) correlated weakly (r_s_ = − 0.17) with Aβ42 (Median 534 pg/mL), but not with tau (Median 216 pg/mL) nor p-tau (Median 31 pg/mL). In a subgroup analysis, the correlation between VV and Aβ42 was only present in the male group (r_s_ = − 0.22, p = 0.038). Further, Aβ42 correlated positively with tau (r_s_ = 0.30, p = 0.004) and p-tau (r_s_ = 0.26, p = 0.012) in males but not in females.

**Conclusions:**

The findings did not support a major dilution effect in iNPH, at least not in females. The only result in favor for dilution was a weak negative correlation between VV and Aβ42 but not with the other lumbar CSF biomarkers. The different results between males and females suggest that future investigations of the CSF pattern in iNPH would gain from sex-based subgroup analysis.

## Background

Idiopathic normal pressure hydrocephalus (iNPH) is characterized by impairment of balance, gait, and cognition as well as urinary incontinence [1, 2]. The cerebral ventricles are enlarged and deformed [3]. INPH is more common than previously thought with a prevalence of 3.7% in the general population above 65 years [4]. The only effective treatment is shunt surgery to divert cerebrospinal fluid (CSF), with 70–80% of patients improving afterwards [5]. The CSF composition differ between patients with iNPH, Alzheimer’s Disease (AD) and healthy controls (HC). The CSF levels of amyloid β 1–42 **(**Aβ42) have been reported to be lower in iNPH than in HC. Accordingly, iNPH patients had decreased or similar levels of tau and phosphorylated tau (p-tau) compared with HC [6–12]. In the same material, but compared with AD, patients with iNPH had lower CSF levels of tau/p-tau but not Aβ42. Current explanatory theories for the lower levels of CSF biomarkers in iNPH are: reduced periventricular metabolism [7, 13], reduced drainage of metabolites from the interstitial fluid to the CSF for example through impairment in the glymphatic system [14–18] or dilution of a fixed amount of biomarkers into a larger than normal CSF volume [19–21].

In perioperative brain biopsies of iNPH patients, concurrent AD pathology has been found in 19–60% [21–25] and a recent study has shown an association between AD pathology on biopsies and lumbar CSF concentration of Aβ42 and p-tau in a cohort of iNPH patients [21]. In AD there are known differences between the sexes. Females have an increased risk of developing AD especially in the highest age groups, even more than can be explained by increased survival compared to males [26–30]. Females with AD or mild cognitive impairment have a higher atrophy rate [31] and suffer a faster disease progression rate [32]. In a cognitively healthy population, no relationship was seen between gender and tau/p-tau while a complex relationship between age, gender and APO-E carriership was seen for Aβ42 [33]. In iNPH, less is known about potential sex differences and although a few iNPH studies include sex-matched controls, subgroup analysis and comparisons are rare. This leaves the question of sex differences in the pathophysiology of iNPH largely unanswered, a question of special interest when investigating Aβ42, tau and p-tau due to the sex differences in AD and possible overlap between the diseases.

The volume of intracranial CSF can be measured by magnetic resonance imaging (MRI), with different methods available from manual to automatic segmentation of the images [34–36]. A reduction in VV have been reported after shunt surgery and clinical symptoms have been seen to correlate with the reduction in volume, implicating the volume change in the pathophysiology of the disease [37, 38]. Given the hypothesis of dilution of the biomarkers in a larger amount of CSF [19, 20], a negative correlation is expected between VV and the concentration Aβ42, tau and p-tau. The objective of this study was to investigate whether such a correlation exists in an attempt to shed further light on the pathophysiology of iNPH.

## Method

### Participants

One hundred and thirty-six patients (47 females and 89 males with a median age of 76 and 75 years, respectively) who underwent surgery due to possible or probable iNPH [39] were consecutively recruited from Sahlgrenska University Hospital (n = 108) during 2013–2016 and Östersund Regional Hospital (n = 28) during 2017–2020. The mean time between imaging and CSF collection was 38.3 days (SD = 72.8). Two patients were excluded because of > 1 year between imaging and lumbar puncture.

A positive shunt response was defined as at least one of the following (a – c) [40, 41]:

a. A 20% reduction in the time or the number of steps in at least one of the two motor function tests (Timed Up and Go, and 10 m walk).

b. Four points increase in the Mini-Mental State Examination.

c. One level increase in the continence scale and two points increase in the Mini-Mental State Examination.

### Ventricular volumetry

**Fig. 1:**
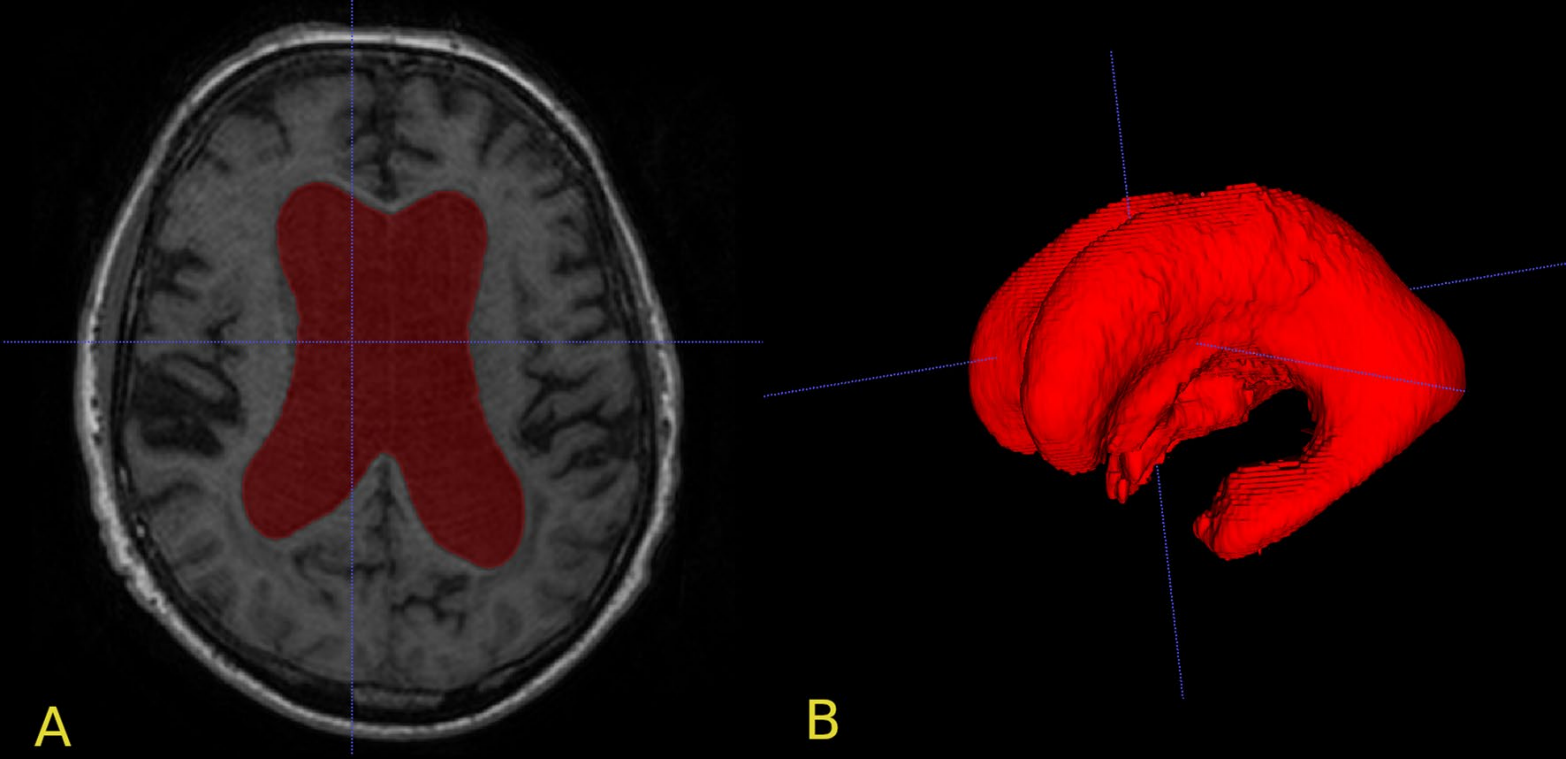
Segmentation example. Example image of the segmentation process using ITK-SNAP. **A** Axial view of the segmentation. **B** 3D reconstruction of the segmented volume

MRI images were acquired before operation with the resolution of 1mm^3^/voxel. The MRI used at Sahlgrenska University hospital was either a 1.5 T Intera (Philips Medical Systems, Best, The Netherlands) or a 1.5 T Achieva dStream (Philips Medical Systems, Best, The Netherlands) whereas the MRI used at Östersund Regional Hospital was either a 1.5 T GE Optima MR450w (GE Healthcare, Wausheka, WI, USA) or a 1.5 T GE Signa Voyager (GE Healthcare, Wausheka, WI, USA). The images were exported using DICOM format. The lateral and third ventricle volumes were semi-automatically segmented using the ITK-SNAP software [34], a method used in a recent study of volumetric change after shunt surgery [42] and exemplified in fig. 1.

### Analysis of CSF

Analyses of Aβ42, tau and p-tau were performed as part of clinical routine workup and the results were retrieved from the participants’ patient charts. Like most Swedish hospitals, both the hospitals in Gothenburg and Östersund purchase this service from the clinical Neurochemistry Laboratory at Sahlgrenska University Hospital, Mölndal, Sweden. CSF was collected in polypropylene tubes during the diagnostic tap test after discarding the initial 3-5 mL to avoid contamination of the sample by small initial bleeding, then centrifuged at 2000 g for 10 min before being frozen at − 70 °C awaiting transportation. Biochemical analysis was performed by the standard clinical procedures at the laboratory using commercially available kits for quantitative solid-phase enzyme immunoassay: INNOTEST hTAU Ag, INNOTEST PHOSPHO-TAU(181P), INNOTEST β-AMYLOID (Fujirebio Europe N.V, Gent, Belgium).

### Statistical analysis

Spearman's rank order correlation was used to analyze associations due to non-normal distribution of the data and the presence of outliers. Confidence intervals for spearman’s rho were acquired by bootstrapping with 3000 samples using the bias correction accelerated method [43]. Between-group comparisons were made using the Mann–Whitney U Test. One participant had a tau value of < 75 pg/mL which was included in the statistical analysis as the value 75 pg/mL. Because of the non-parametric tests used and it being the single lowest value in the data set this should not have impacted the reliability of results. The level of statistical significance was set to p < 0.05. Statistical analysis was performed using R version 4.0.3 (R Foundation for Statistical Computing, Vienna, Austria). Power calculation was made using G*Power 3.1.9.4. [44]. The sample size was large enough to have discovered correlations of effect size 0.3 or larger with 95.4% power, per Cohen’s convention a medium effect [45].

## Results

**Fig. 2:**
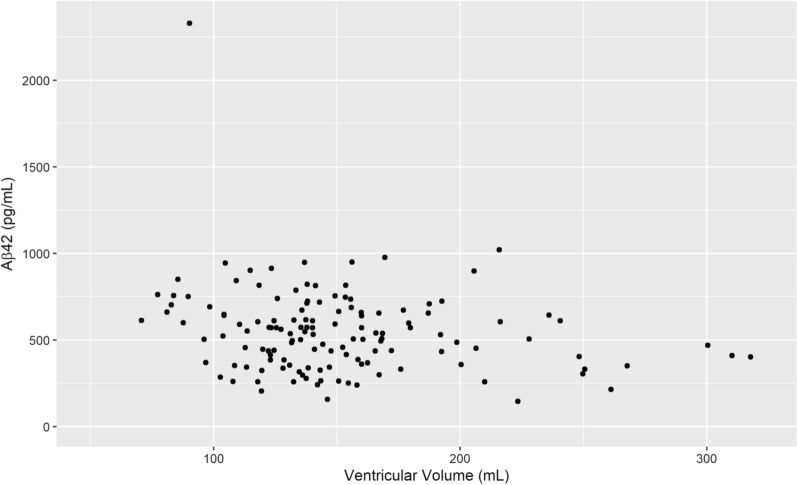
Ventricular Volume and Aβ42 concentration. Scatterplot of ventricular volume Aβ42 concentration for the full sample, n = 136

**Fig. 3:**
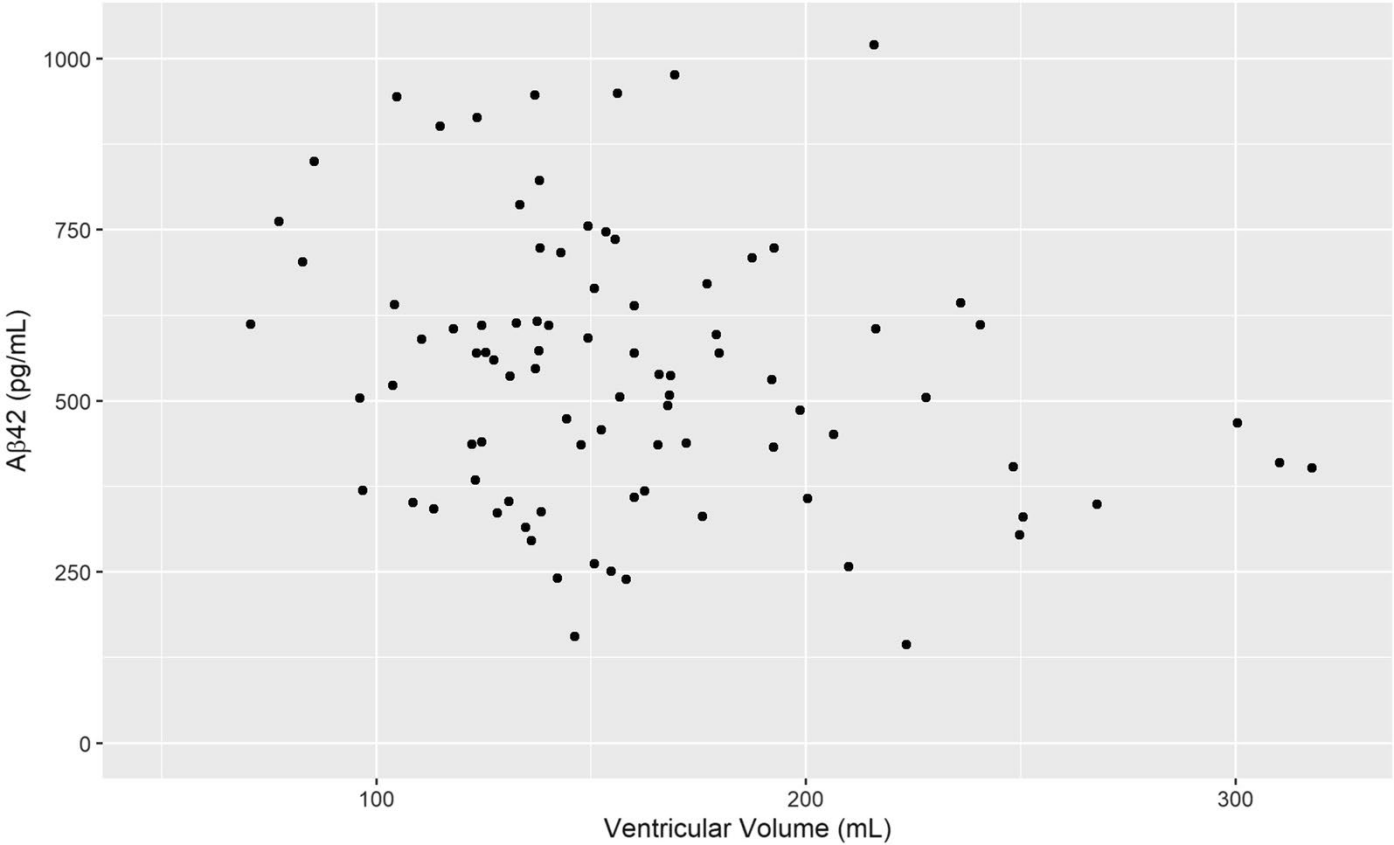
Ventricular Volume and Aβ42 concentration in the male subgroup. Scatterplot of ventricular volume Aβ42 concentration for the male subgroup, n = 89

**Fig. 4:**
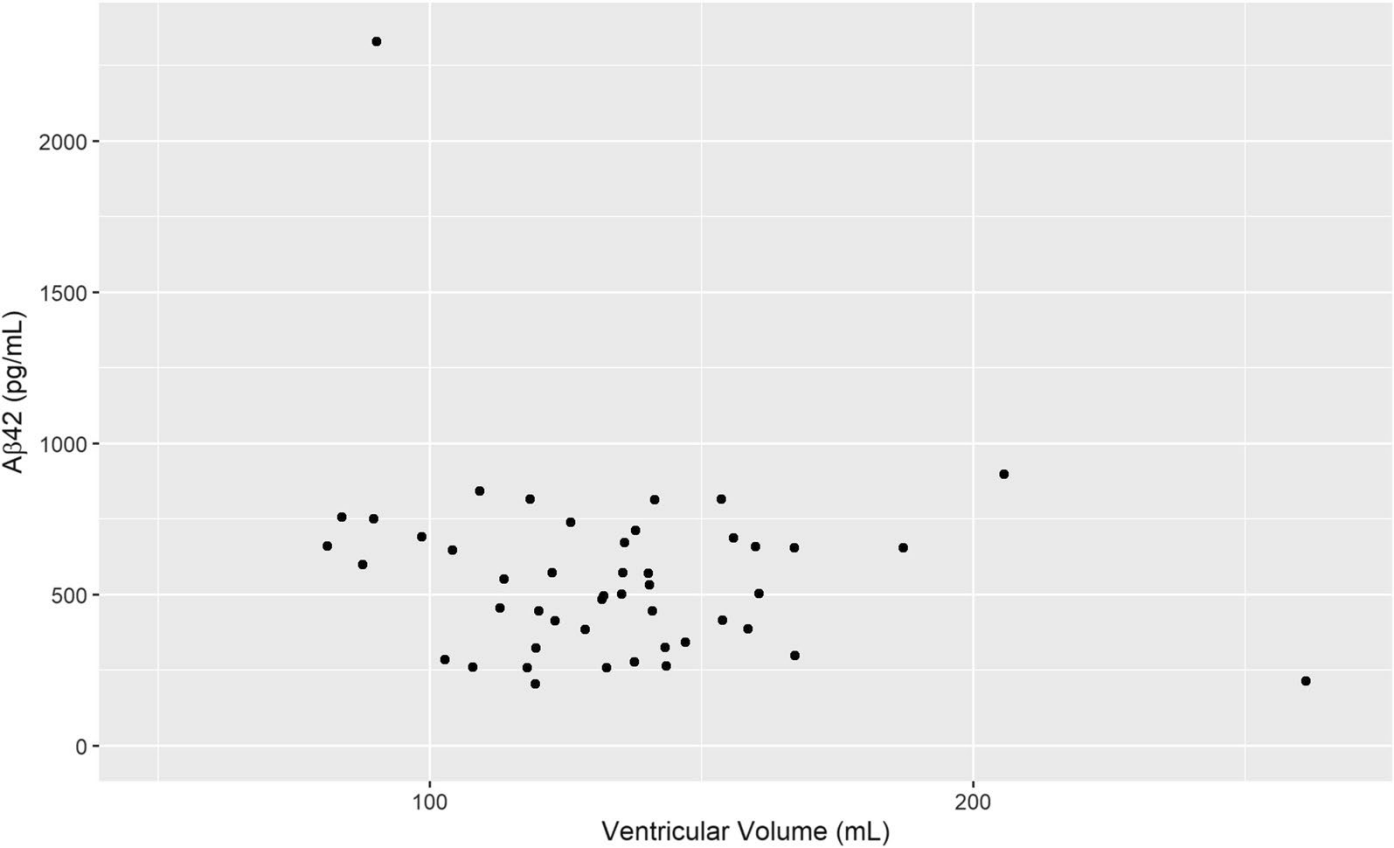
Ventricular Volume and Aβ42 concentration in the female subgroup. Scatterplot of ventricular volume Aβ42 concentration for the female subgroup, n = 47

**Fig. 5:**
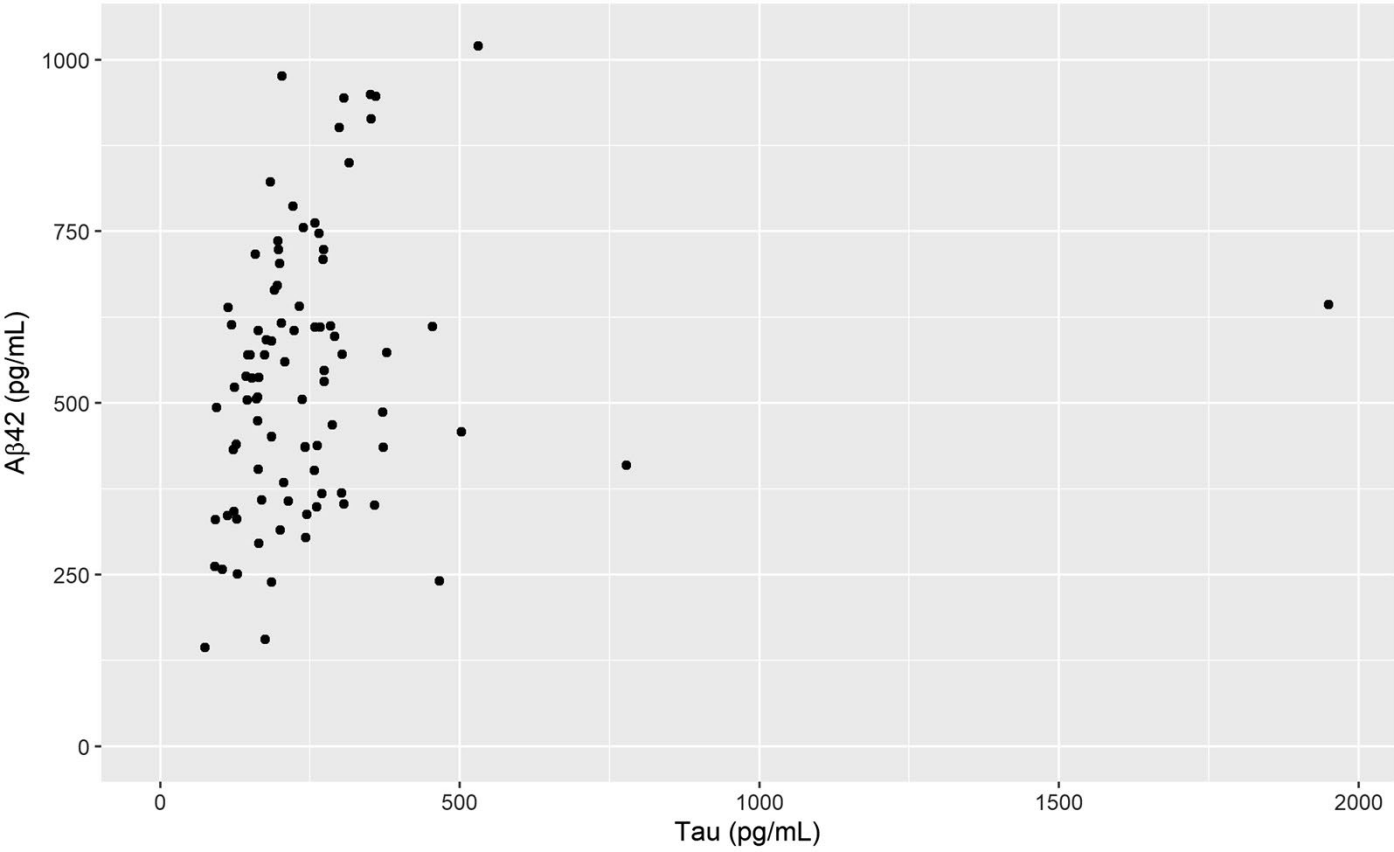
Tau and Aβ42 in the male subgroup. Scatterplot of tau and Aβ42 concentration for the male subgroup, n = 89

**Fig. 6:**
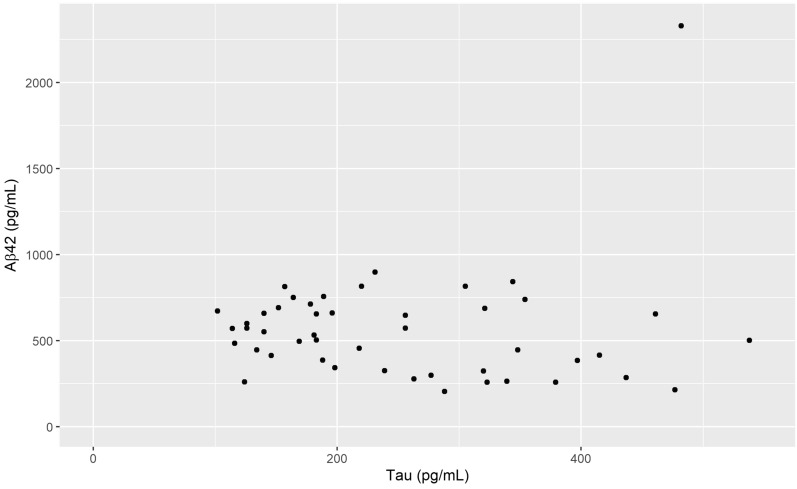
Tau and Aβ42 in the female subgroup. Caption: Scatterplot of tau and Aβ42 concentration for the female subgroup, n = 47

For the total sample (n = 136) VV had a negative correlation with Aβ42 (r_s_ = − 0.17, p = 0.042) which is visualized in Fig. 2, but not with tau or p-tau. However, the correlation between Aβ42 and VV was slightly stronger in the male subgroup (r_s_ = − 0.22, p = 0.039), visualized in Fig. 3. For males there was also a positive correlation between Aβ42 and tau (r_s_ = 0.302 p = 0.004) as well as p-tau (r_s_ = 0.264 p = 0.001). In females, no significant correlation between VV and the CSF biomarkers was found (Table 1). The relationship between Aβ42 and VV for females is shown in Fig. 4 and the interrelationship of the biomarkers for the subgroups in Fig. 5 and 6.

**Table 1:** Correlation between ventricular volume, Aβ42, tau and p-tau

	Ventricular Volume	Aβ42
Full sample (n = 136)		
Aβ42	− 0.17* (− 0.33, 0.0028, p = 0.042)	
Tau	0.006 (− 0.16, 0.18, p = 0.94)	0.10 (− 0.073, 0.28, p = 0.22)
p-Tau	− 0.014 (− 0.18, 0.16, p = 0.86)	0.14 (− 0.032, 0.31, p = 0.11)
Males (n = 89)		
Aβ42	− 0.22* (− 0.40, − 0.022, p = 0.038)	
Tau	− 0.042 (− 0.24, 0.19, p = 0.70)	0.30* (0.088, 0.50, p = 0.004)
p-Tau	< 0.001 (− 0.24, 0.23, p = 0.99)	0.26* (0.051, 0.45, p = 0.012)
Females (n = 47)		
Aβ42	− 0.12 (− 0.43, 0.18, p = 0.40)	
Tau	0.15 (− 0.15, 0.41, p = 0.31)	− 0.19 (− 0.44, 0.14, p = 0.21)
p-Tau	0.038 (− 0.25, 0.31, p = 0.80)	− 0.054 (− 0.31, 0.26, p = 0.71)

Rho values for Spearman’s ranked order correlation. Within parenthesis 95% confidence intervals for Spearman’s rho and p-value. *Significant results

Median VV and CSF biomarker concentrations are shown in Table 2. Males had significantly larger VV than females, but no significant differences were found in CSF biomarker concentration, age nor in improvement rate. Neither did the two clinics differ significantly in these variables nor in sex distribution. Detailed clinical information was available for a subset of the participants consisting of 83 individuals. At the first follow-up, 63 (76%) had improved after surgery.

**Table 2:** Participant characteristics

	Total Sample (n = 136)	Males (n = 89)	Females (n = 47)
Age (years)	76 (Q1 71, Q3 79)	75 (Q1 71, Q3 79)	76 (Q1 72, Q3 79)
MMSE (presurgical)	26 (Q1 22, Q3 28, n = 83)	26 (Q1 22, Q3 28, n = 47)	25 (Q1 23, Q3 28, n = 26)
TUG time (seconds, presurgical)	17 (Q1 11.9, Q3 26.0, n = 83)	16 (Q1 11.0, Q3 26.0, n = 47)	18.3 (Q1 13.5, Q3 26.0, n = 26)
Improved	63 (75.9%, n = 83)	45 (79%, n = 47)	18 (69%, n = 26)
VV	141 mL (Q1 123, Q3 166)	151 mL (Q1 128, Q3 179)	133 mL (Q1 112, Q3 142)
Aβ42	534 pg/mL (Q1 380, Q3 662)	536 pg/mL (Q1 384, Q3 641)	532 pg/mL (Q1 364, Q3 680)
Tau	216 pg/mL (Q1 163, Q3 300)	208 pg/mL (Q1 163, Q3 274)	220 pg/mL (Q1 160, Q3 331)
p-tau	31 pg/mL (Q1 23, Q3 40)	30 pg/mL (Q1 23, Q3 38)	33 pg/mL (Q1 24, Q3 45)

*VV* Ventricular volume, *Q1* first quartile, *Q3* third quartile, *MMSE* Mini Mental State Examination, *TUG* Time Up and Go test

## Discussion

Considering previous reports of generally low CSF concentrations of Aβ42, tau and p-tau in iNPH [6–12] we aimed to detect a possible dilution effect with the use of MRI volumetry. To our knowledge this has not been done previously. In our total sample of 136 patients with iNPH there was a correlation between VV and Aβ42 though no correlation between VV and t-tau nor p-tau. Interestingly, this correlation seems carried by the male subgroup. Considering the low effect size and isolated significance regarding VV, our results do not support dilution as the major cause of lower levels of CSF biomarkers reported in iNPH. The levels of Aβ42, tau and p-tau were similar to previous studies on iNPH patients [8, 10, 11], though slightly higher than in others [7, 19, 46, 47]. The total shunt response rate of 76% was also in level with previous reports [5]. In agreement with our findings, Tullberg et al. [48], reported no correlation between the lateral ventricle area, and the CSF concentration of albumin, NFL, Tau and several other compounds. However, in contrast to the latter study, we used three-dimensional volumetric measurement of the cerebral ventricles.

In the present study, the relation between VV and CSF biomarkers levels in iNPH patients differed according to sex. In males, but not in females, we found a negative correlation between VV and levels of Aβ42 which is in line with previous findings in mild cognitive impairment and AD [49, 50]. An unexpected finding was the positive correlations between Aβ42 and tau as well as p-tau in the male subgroup in contrast to an expected negative correlation given the increasing prevalence of AD with age and the associated effect on Aβ42, t-tau and p-tau [21]. In preclinical AD, CSF biomarkers are known to change years before cognitive decline [51]. A higher prevalence of AD has been shown in females [26–30] and a high comorbidity between AD pathology and iNPH has been found in perioperative brain biopsies [52, 53]. With this in mind, one possible explanation for our discrepant findings between the sexes could be a higher prevalence of preclinical AD pathology in the female group. However, this explanation is contradicted by the lack of difference between the sexes regarding absolute concentration of Aβ42, t-tau and p-tau. Another explanation is that there are sex differences in the complex pathophysiology of iNPH affecting the pattern of CSF-biomarkers. In a recent study, Graff-Radford et al. [54] underlined the need to take CSF disorders like iNPH into account in the interpretation of Aβ42, tau and p-tau levels. Further studies on a possible dilution effect would gain from complementary information on AD pathology, for example APO-E genotype and AD pathology in perioperative brain biopsies, to explore interaction between the effects of sex, iNPH and AD on CSF biomarker patterns.

In a previous study, an increase in Aβ42 has been observed in patients with iNPH one year after shunt surgery, though only in a low tau-subgroup [55]. In the high tau-subgroup, Aβ42 remained unchanged. Due to a cognitive decline in the high tau group the authors considered the possibility of AD pathology in the group, this could in theory cause a progressive lowering of Aβ42 canceling out the effects of shunting at follow-up. A recent longitudinal study on CSF biomarkers before and after shunt surgery found a postsurgical decrease in Aβ42 and simultaneous increase in tau, p-tau [52]. The authors argued that decrease in Aβ42 was mainly driven by APOE epsilon 4 carriers, indicating a possible role of AD pathology in the evolution of biomarker concentrations. In other studies, an increase in the levels of different lumbar CSF biomarkers was seen after shunt insertion, except for Aβ42 that was unchanged [8, 56]. The latter findings seem to contradict the dilution theory, given that the expected outcome of a decrease in VV following shunting would be an overall increase in the concentrations of CSF biomarkers, including Aβ42. However, lumbar CSF composition of brain-derived proteins is affected by absorption and metabolism along the spinal canal [57] and as such it susceptible to the changes in fluid dynamics introduced by a shunt and effects of surgery on CSF content should be interpreted with caution.

In summary, our results do not support dilution to be the major mechanism behind the lower lumbar CSF concentrations of Aβ42, t-tau and p-tau found in iNPH. Due to the unexplained sex differences, however, the possibility of a minor dilution effect cannot be dismissed. As our study focused on whether dilution could explain the lower concentrations of Aβ42, tau and p-tau in iNPH, our results provide no further insight regarding other possible pathophysiological explanations.

### Strengths and limitations

That our material consists of patients undergoing routine clinical care for iNPH with a shunt response rate and levels of Aβ42, tau and p-tau in accordance with previous reports supports generalizability. Lumbar CSF was used which is a methodological limitation shared with most previous studies on CSF composition in iNPH. Levels of biomarkers of ventricular CSF would probably have been more relevant to relate with VV and better reflect the pathophysiology of iNPH. While there is a known difference between ventricular and lumbar CSF composition [48, 57], how predictable the difference is is largely unknown. One study showed a strong correlation between ventricular and lumbar CSF composition, although based on only five participants [58]. Further research in this area would be valuable, however ventricular CSF from healthy individuals is seldom available.

In our study, neither the spinal nor other extraventricular CSF was covered by the volumetric measurements. As the largest increase in CSF volume in iNPH is intraventricular this is unlikely to have masked a dilution effect.

The material provided sufficient power to detect a medium or larger effect supporting its reliability. For the subgroup analyses the power was weaker, though considering the small overlap between the confidence intervals of r_s_ for the correlation between Aβ42 and tau in particular, a true difference between the subgroups is likely to exist.

## Conclusion

A major dilution effect was not found when analyzing the correlation between VV and lumbar CSF levels of Aβ42, tau and p-tau biomarker in the total sample of 136 iNPH patients. Subgroup analyses revealed sex-based differences which highlights a need for stratification when investigating iNPH pathophysiology.

## Data Availability

The data that support the findings of this study are available on request from the corresponding author after appropriate ethical review board approval.

## Abbreviations

AD: Alzheimer's Disease
Aβ42: Amyloid-β 1–42
CSF: Cerebrospinal fluid
HC: Healthy Controls
iNPH: Idiopathic normal pressure hydrocephalus
MRI: Magnetic resonance imaging
Md: Median
P-tau: Phosphorylated tau
SD: Standard Deviation
VV: Ventricular volume
